# Light Pollution Monitoring and Sky Colours

**DOI:** 10.3390/jimaging6100104

**Published:** 2020-10-05

**Authors:** Zoltán Kolláth, Dénes Száz, Kornél Kolláth, Kai Pong Tong

**Affiliations:** 1 Department of Physics, Eötvös Loránd University (ELTE) BDPK, 9700 Szombathely, Hungary; szaz.denes@gmail.com (D.S.); kollath.k@met.hu (K.K.); tong.kai.pong@sek.elte.hu (K.P.T.); 2 Hungarian Meteorological Service, 1024 Budapest, Hungary

**Keywords:** light pollution, imaging radiometry, colorimetry

## Abstract

The measurement of night sky quality has become an important task in nature conservation. The primary device used for this task can be a calibrated digital camera. In addition, colour information can be derived from sky photography. In this paper, we provide a test on a concept to gather information about the possible sources of night sky brightness based on digital camera images. This method helps to understand changes in night sky quality due to natural and artificial changes in the environment. We demonstrate that a well-defined colour–colour diagram can differentiate between the different natural and artificial sources of night sky radiance. The colour information can be essential when interpreting long-term evolution of light pollution measurements.

## 1. Introduction

Light pollution, caused by the increasing level of artificial light at night (ALAN), has become a significant environmental problem in the last few decades. With accelerating urbanisation, the area affected by ALAN and the brightness of light pollution is growing annually [1]. Artificial lights influence the natural behaviour of nocturnal animals by making them disoriented and misled by additional illumination, which affects foraging, reproduction, communication, and other critical behavioral patterns [2,3,4,5,6]. With the mechanism of altering the circadian cycle and inhibiting melatonin production in several species, including human, light pollution has adverse impacts on health and the natural quality of life [7,8,9,10]. To reduce the impact of night lighting, the development of new lighting strategies is necessary that minimizes adverse ecological impacts while providing sufficient light for human needs and safety [11]. One progressive step towards the optimisation between light requirements and environmental impact has been realized in the areas of Zselic and Bükk starry sky parks in Hungary, where the whole lighting system of two settlements has been reconstructed based on a special new design of LED lamps [12]. To determine the extent of light pollution at a given location, reliable measurement techniques and continuous monitoring are required.

The widely used single-channel measurement devices, e.g., the Sky Quality Meter (SQM), have several drawbacks for measuring light pollution. The SQM (e.g., [13]) has a custom filter that does not exactly match any astronomical or photopic band, but has an extra sensitivity at blue wavelengths. The displayed unit is often not compatible with the standard astronomical definition of magnitude, since the reference is given by a stellar spectrum which has a different level at different wavelengths.

For multi-channel measurements, commercially available digital single lens reflex (DSLR) and mirror-less (MILC) cameras provide a viable opportunity to monitor the quality of the night sky and light pollution [14]. These cameras can save images in raw format, can be calibrated to measure the radiance of the sky, and the distribution of sky brightness can be represented by using false colour images [15,16,17,18,19]. Digital cameras are usually used for all-sky measurements with fish-eye lenses. Images taken with all-sky cameras have a good resolution near the zenith and most parts of the sky, however, they lack the resolution and precision close to the horizon, which is the region of the sky that is normally the most interesting for light pollution research. This problem can be solved by taking two or multiple fish-eye images in the vertical plane [20], although, then, the resolution of the zenith becomes poorer. The highest precision and resolution at dark locations and under clear sky conditions can be achieved with a robotic panorama head with a 24 mm or 35 mm rectilinear lens on a full-frame digital camera [21]. With this set-up, the camera covers the whole sky and some of the ground and environment with 28 individual images taken at different pointing directions with high spatial resolution. Using 6–10 s exposure times, a whole sky image can be acquired in 10–15 min of measurement time at a given location. With short exposure time, star trails and the apparent rotation of the sky do not cause artefacts during the measurement. Post-processing corrections of the images are also available based on astrometry [21].

Today, there are also commercially available solutions for image processing with DSLR cameras to measure sky quality, such as the “Sky Quality Camera” (SQC) software (Euromix, Ljubljana, Slovenia) [22,23]. The camera is photometrically calibrated by the software manufacturer. They are using the green channel of the chip for photometric calibration which also includes corrections of optical aberrations like vignetting. The software provides the luminance for each pixel, and it is possible to calculate the illuminance from the luminance data, as well as the CCT from the three colour channels. The drawback of the system is the high price, and, if you want to use the system, you either have to buy a pre-calibrated camera, or you have to send your own to the manufacturer for calibration. A similar alternative is the systems of the German Technoteam corporation who provide a photometry solution (digital camera and software) commercially (https://www.technoteam.de/product_overview/photometer_colorimeter/products/lmk_mobile_air/index_eng.html). This system is able to create high-resolution luminance data and statistics that can be evaluated in MsExcel, MatLAB, and LabVIEW. The system has the restrictions that it cannot be used for measuring coloured light sources (i.e., LED) and measuring modulated light sources with a high depth of modulation is limited. Instead of these systems, we preferred to use our self-developed open-source alternative, DiCaLum, that works similarly and the data of which can be further analyzed, and additional information can be obtained from other spectral channels.

Digital cameras have already been used to estimate radiance in different colour bands. The imagery obtained from the International Space Station is used to estimate the spectral type of ground light sources based on colour information [24]. The colour information of DSLR cameras is also used to estimate human photoreceptoral inputs based on the spectra of standard light sources [25]. There is an increasing interest in colour detection based on single detector pointing devices as well, see, e.g., [26] and the CoSQM project (https://lx02.cegepsherbrooke.qc.ca/~aubema/index.php/Prof/Cosqm-users-manual).

Light pollution is not always easy to separate from the spectrum of the night sky. Even the clear natural night sky spectrum with no light pollution can be very different based on various factors such as geographical location, air conditions, meteorological conditions, etc. All physical bodies and particles in the Earth’s atmosphere have the ability to reflect, absorb, and scatter light. Clouds can reflect light to the scattered light causing the radiation reaching the surface and adding another component to the sky brightness [27]. The elevated level of sky brightness near urban areas under clouds can be measured by all-sky photometry even from several km distance from the city [28]. To have a reliable method of monitoring light pollution, it is important to use an appropriately calibrated device for measurement, to have knowledge on the natural sky spectrum and to use an SI traceable unit for dark sky characterization.

The existing measurement methods are calibrated by different methods using different reference targets. Therefore, the metrics and units used by different research groups are not unified. Moreover, these units are not necessarily fully compatible with the standard definitions. It is common to use a standard source or a calibration device with spectral characteristics which differ from the spectral response of the measurement device. Digital cameras are calibrated by astronomical or standard CIE photometry [29]. In [21], we suggested a measurement method based on the calibration of digital cameras with known narrow-band light sources and a spectroradiometer with which the natural sky spectrum could be precisely determined as well. We introduced a new metric, the dark sky unit (dsu), which is an SI traceable unit (nW/m${}^{2}$/sr/nm) for measuring sky brightness and can be determined separately for the three colour channels of the digital camera. The measurement method took into consideration the natural changes in the sky radiance, like airglow, the natural emission of the molecules, and atoms in the upper atmosphere that cause dynamic changes in the night sky radiance, especially in places with negligible light pollution.

In this paper, we demonstrate a method for sky quality analysis based on the real colour information of the night sky measured with digital cameras using the method of [21]. The main difference compared to previous works is that, in this paper, we use the spectra of the natural sky to define colour transformations as metrics.

## 2. The Colour of the Night Sky and Its Measurements

The digital cameras (DSLR or MILC) provide an opportunity to measure the radiance of the night sky. We recently introduced a new metric to determine night sky quality [21]. The recommended metric is band-averaged spectral radiance with a unit of nW/m${}^{2}$/sr/nm which is abbreviated as dsu (Dark Sky Unit). The red, green, and blue channels provide three band-averaged spectral radiance values: ${\bar{L}}_{R}$, ${\bar{L}}_{G}$ and ${\bar{L}}_{B}$. To determine the colours, these quantities can be normalized to get $\ell_{X}=c\times {\bar{L}}_{X}$ and $\ell_{R}+\ell_{G}+\ell_{B}=1$. With this normalisation ${\bar{L}}_{G}$, $\ell_{R}$ and $\ell_{G}$ provide a full description of the digital camera measurement of skyglow at a given direction.

**Figure 1:**
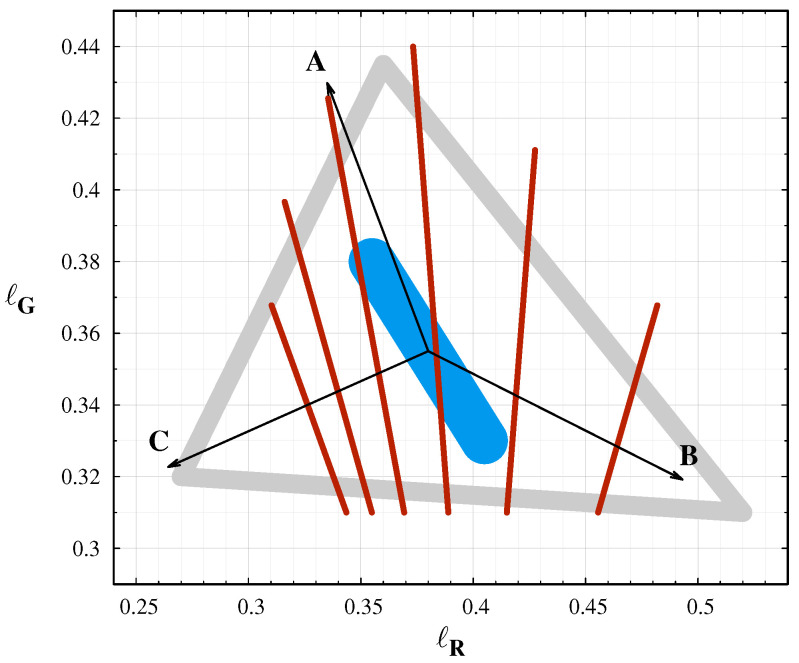
The possible colour range of natural night sky colours. The most probable colours are inside the grey triangle. The cyan band indicates the standard sky with no extreme airglow and with no light pollution. The arrows indicated the possible shifts by different sources: A: Green oxygen airglow, B: Sodium airglow, C: Twilight. The red lines indicate the approximate equi-CCT curves with CCT = 5500, 5000, 4500, 4000, 3500 and 3000 K (from **left** to **right**).

In a companion paper [30], we analyzed the possible colours of the natural night sky. Figure 1 displays a schematic view of the possible night sky colours. The different components of sky radiance together allow only a limited range of colours, ranging among orange and green. The natural green radiation of the sky shifts the neutral night sky colours to the green, while sodium or red oxygen emission makes the sky more orange. The spectrum of the night sky contains the fingerprints of the different light sources. Therefore, spectroradiometry provides the most complete information about sky radiance.

We performed an extended spectral survey in the Zselic Dark Sky Park, Hungary. In [21], we already demonstrated that the spectrum can be perfectly fitted by the natural sky model and the spectrum of the light sources in the neighbouring settlements.

**Figure 2:**
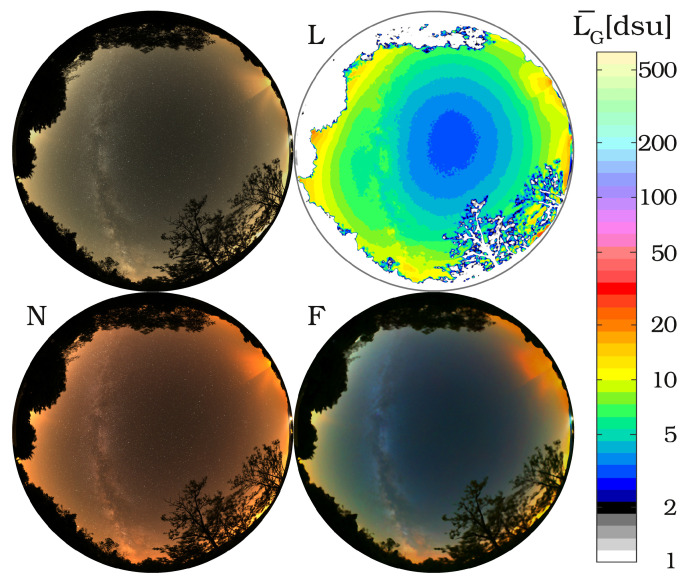
Radiance and colour analysis of an all-sky image. RGB image with dsu colours (**top left**); L: Radiance map in G band; N: real colour representation; F: colour enhanced photo to emphasize the different sources.

Figure 2 shows an example for different colour representations of the night sky. We used dsu based colours to create the RGB image from the RAW image of the camera shown in the top left corner. Typically, we use the green band of the camera to compute the radiance map illustrated with false colours in dsu units, as shown in the top right. For colour analysis of the image, it is essential to know the real colours of the night sky. In the bottom left corner, the image is presented in real colours. The bright bluish images of astrophotographers are often deceiving, since they manually enhance the colours to make them more spectacular, but they also become less realistic. We also used a specific colour enhancement based on a well defined colour conversion that can emphasize the different light polluting sources, seen in the bottom right. The method of conversion between colour spaces is described in [30].

## 3. Colour Analysis of All Sky Images

We routinely monitor sky quality at different locations, primarily in Hungarian national parks, but also at worldwide locations. Here, we selected data from places with no light pollution or minimal artificial light at night (ALAN) present. In addition, we selected observations where the natural radiation of the sky in visual bands were extreme. We obtained natural sky data in Ontario, Canada (coordinates: 48.4745°, −81.7577°) on 25 August 2019 at 4:02 a.m. UT. The measurement at the Cosmic Campground Dark Sky Sanctuary (coordinates: 33.4796°,−108.9228°) on 20 October 2019 at 3:53 a.m. UT are strongly influenced by a strong sodium event [21]. Two days after the Cosmic Campground measurements, we detected less sodium radiation but still an intense green oxygen airglow at another place in New Mexico, USA, at the El Malpais national Monuments (coordinates: 34.9609°, −108.1301° on 22 October 2019 at 5:30 a.m. UT). In the Zselic Landscape Protection Area in Hungary (which coincides with the Zselic Starry Sky Park), we perform measurements routinely. We selected the darkest night we have encountered there (coordinates: 46.2697°, 17.6621° on 19 April 2018 at 3:00 a.m. UT).

**Figure 3:**
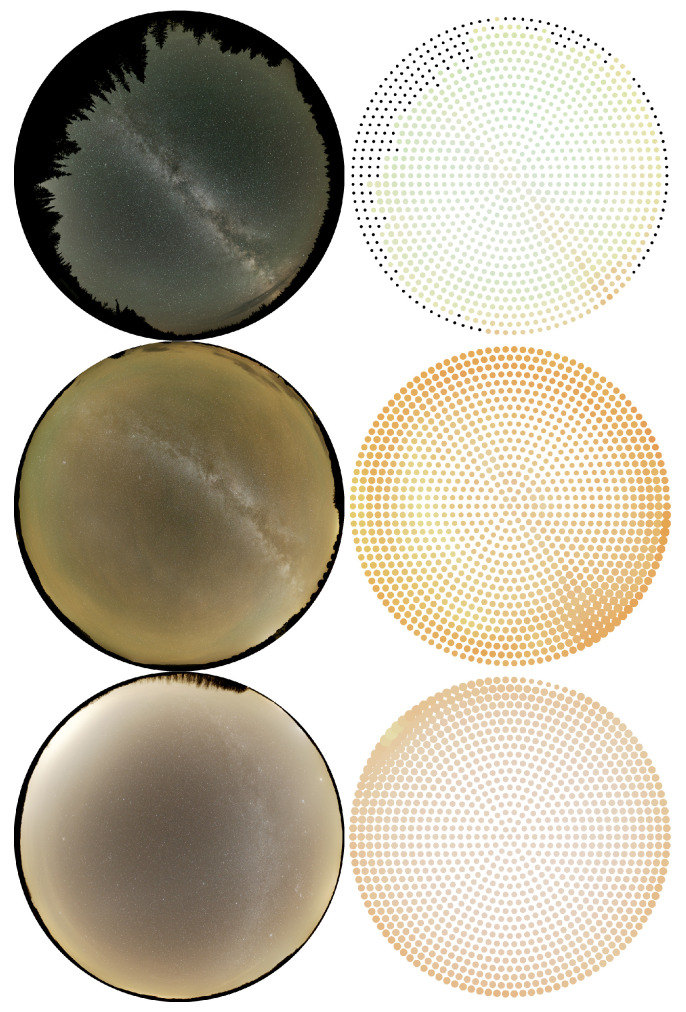
All-sky images and and their colour representation.

**Figure 4:**
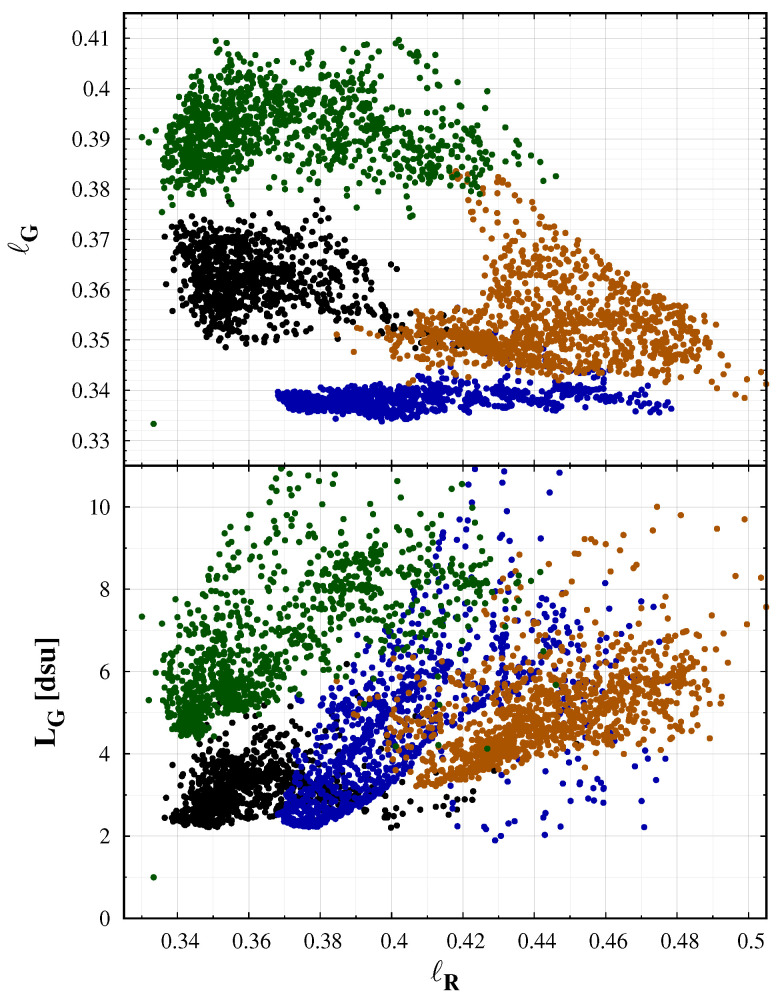
Colour–colour (**top**) and colour–radiance (**bottom**) diagram calculated from all-sky images taken at worldwide locations. Black: remote location in Canada, natural sky; Green: El Malpais National Monument, NM, USA, some light pollution, strong green airglow; Orange: Cosmic Campground Dark Sky Sanctuary, NM, USA, no ALAN, strong sodium airglow; Blue: Zselic, some medium level ALAN, best night with data.

For colour analysis, we first calculated the dsu colour image, then we defined a grid along horizontal and azimuthal angle with a high enough arbitrary resolution and determined the colour value based on the dsu colour indices in each grid point. Thus, we got the colour representation of all-sky images as shown in Figure 3. The obtained colour dataset can be displayed in a colour space defined by the red and green colour indices. In Figure 4, the diagram on the top shows the data of the above-mentioned locations. It is clearly visible that, in New Mexico (El Malpais), where the green airglow was strong, the data points are shifted toward higher $\ell_{G}$ values. Similarly, the data of Cosmic Campground Dark Sky Sanctuary are shifted towards higher $\ell_{R}$ values that reflect the sodium airglow measured at the location. Another representation of these data is when the green band colour radiance is displayed in the function of the red colour index. Combining these two plots, we could estimate the sources of sky brightness based on the colour and radiance distribution of the measured night sky. The dispersion of the point from a given location is a result of the different zenith distance and/or the different level of airglow. In the Cosmic Campground data-set, the increasing $\ell_{R}$ content correlates with the radiance as expected. In the case of the Zselic measurements, the green content ($\ell_{R}$) has a very narrow range, indicating a low airglow level. However, close to the horizon, the level of light pollution component is increased, which results in increased $\ell_{R}$ and radiance level. The most compact scenario is obtained at the Ontario, Canada location where both the airglow and the light pollution level were low. Then, the dependence on the sky coordinates, especially on the zenith distance, is less prominent.

## 4. Long-Term Monitoring of Light Pollution by Digital Cameras

A good method of monitoring the temporal changes of sky quality and night sky colour of a specific location is to install a remotely controllable fixed all-sky digital camera with a fish-eye lens. Our first fixed camera was installed at the Zselic Observatory, in the area of Zselic Starry Sky Park being the first dark sky park in Hungary and Europe. The camera took photos daily every 10 min from sunset until sunrise. Thus, we got a time series for a longer period, based on which the variation and dynamics of airglow can be monitored.

**Figure 5:**
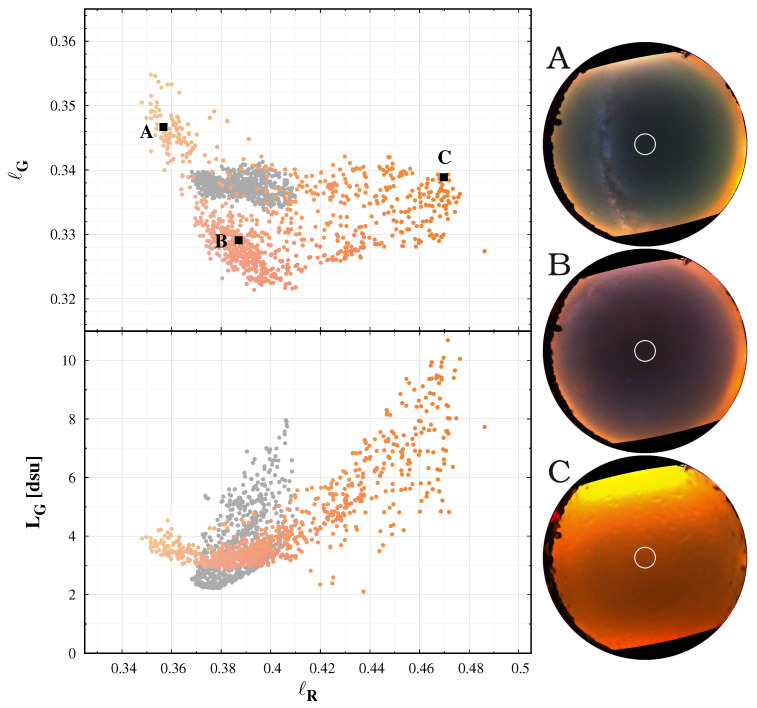
Left: Colour–colour (**top**) and colour–radiance (**bottom**) diagram calculated from the all sky images taken in the Zselic Starry Sky Park at a fixed measurement station. The colours of the symbols represent the real colour of the sky. The grey dots display for comparison the darkest night from a simple image. Right: Three typical all sky images from the permanent camera in Zselic. The white circle indicates the part of the sky used for the analysis. The corresponding colour pairs are indicated by the letters (**A**–**C**).

Figure 5 displays the green band-averaged radiance in dsu units as a function of the red colour index and the colour–colour diagram for all-sky camera measurements of the same location in Zselic during a longer time period from 31 March 2020 to 14 June 2020. The colour of the dots represents the real colours of the sky. For comparison, the central values of the darkest night observed in the Zselic are also displayed with gray colour. A clear lower boundary of data points can be found at $\ell_{R}=0.38$ where the minimum band averaged radiance is $L_{G}=2.7\;\mathrm{dsu}$ which represents the best night at the location with minimal airglow. Moving left from the diagram from the minimum point, the green airglow increases, thus the radiance also increases. At the redder part, the reddish colour refers to an increased level of clouds, which makes the sky brighter.

We emphasise the difference between the regions denoted by ‘A’ and ‘B’. There is a clear separation on the colour–colour ($\ell_{R}-\ell_{g}$) diagram. However, the separation in the radiance level is only marginal, compared to the weather-related changes. It is an important factor when long-term changes are analysed. The evolution of the sky radiance can be affected by the degradation of the instrument. The airglow level depends on solar activity, which varies on multiple timescales. In addition, the changes in the global structure of the atmosphere related to climate change may also induce variation; for example, the tropospheric density strongly affects the airglow levels. A single channel measurement sequence, like the results of the recently standard SQM measurements station, can show the variation in radiance. Still, one needs the colour information—in addition to correctly interpret the origin of the changes. The colour–colour diagrams are crucial tools in this sense.

These differences in sky colour can be visually enhanced if we plot the whole sky images in false colour enhanced (FCE) mode (see about this method in [30] and in the discussion), which is useful for distinguishing the spectral and radiance changes. The right panel in Figure 5 demonstrate the FCE images for three selected colour pairs.

## 5. Discussion

**Figure 6:**
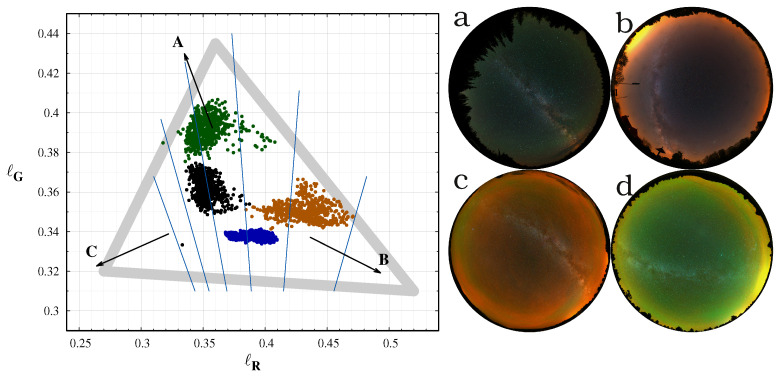
Left: The measured colours from four different situations. The most probable colour range is indicated by the grey triangle. The arrows indicated the possible shifts by different sources: (**A**): Green oxygen airglow, (**B**): Sodium airglow, (**C**): Twilight. Right: false colour enhanced all sky images at the four location: (**a**): Ontario, Canada (black), (**b**): Zselic (blue), (**c**): Cosmic campground (orange), (**d**): El Malpais (green). the colour names in parenthesis gives the symbol colours in the left image. The cyan lines indicates the regions with equal CCT from 3000 K (**right**) to 5500 K (**left**) in 500 K steps.

This paper provides a proof of concept whether camera colours can be used to distinguish different sources of night sky radiance. Figure 6 displays the measurement at four different locations with different sky conditions. Here, we selected only the colour values from the all sky images with at least 30° elevation above the horizon. This selection eliminates the extremely reddened regions due to air scattering and light pollution close to the horizon. In this figure, the different situations are well separated, but all the measurements are inside the expected range of colours. The extreme airglow at the Cosmic Campground and at the El Malpais National Monument shifts the colours according to the theoretical predictions. In the first case, the sodium events shift the colours to the orange. The reduced sodium emission and the increased green oxygen emission at the second case results in a greener sky colour. The sky at the Ontario, Canada location was a natural one, with no light pollution and minimal airglow. Therefore, the colour coordinates are close to the center of the natural colours, with some increase in the green level. The darkest night we observed in the Zselic Dark Sky park was a special one. The airglow level was low, and, at the same time, the atmosphere was clean, free from aerosols and humidity. It resulted in less scattering of the ALAN from the neighbouring city and villages. It represents a natural sky, but still different from the situation encountered in Canada.

As colour information, usually the correlated colour temperature (CCT) is used. However, it is only a one-dimensional representation of the colours, with an underlying assumption that the light-emitting object behaves similarly to a blackbody radiator, the closeness of which is usually quantified by the colour rendering index (CRI). CCT alone cannot distinguish between different levels of airglow, since airglow arises not from blackbody absorption and radiation, but rather from the emission of photons by means of excitation of specific molecules (e.g., oxygen and sodium as shown above) by incoming solar radiation in the ionosphere. The equi-CCT lines in Figure 6 clearly demonstrate this situation. Three of the four samples lie in the 4000–4500 K range. The colour temperature alone cannot separate these different measurements, but the colour–colour diagram defined by the *ℓ* colours clearly display the separation. The colour–colour diagrams and the false colour enhanced images are superior compared to the CCT in describing the constituents of the radiation of the night sky.

In a tricolour system, where the chromaticity coordinates are defined in a two-dimensional plane, there are other possibilities to display the image colours. The primary colour space is the CIE Yxy space; another frequently used system is the CIE L*a*b*. From the camera measurements, there is no exact one-to-one transformation. Still, it is possible to fit an approximate colour-transformation based on a well-defined learning set of possible sky spectra. We tested these approximated xy, and a*b* chromaticity diagrams as parallel diagnostic tools for the separation of different scenarios of sky colours/radiance. According to our recent observational data and theoretical models, these standard colour spaces do not provide any improvement in colour separation compared to the native camera *ℓ* colour values. Therefore, we prefer to use the native camera values since then we do not have to apply any approximate transformation. In addition, the L*a*b* transformation is based on nonlinear functions, and, in the measurements, we prefer to use only linear transformations.

A quantitative test on the goodness of the used colour space is the correlation between the different colour coordinates. The best visualisation is possible with chromaticities which are uncorrelated. For the data-set presented in Figure 6, the correlation between the $\ell_{R}$ and $\ell_{G}$ is only −0.3. It is possible to introduce a colour transformation which minimises the correlation between the chromaticity coordinates by the eigenvectors of the correlation matrix of the data vectors. With the same data, we managed to reduce the correlation, but the visual inspection of the colour plots does not show any significant improvement. Therefore, we recommend using the camera-based natural choice of $\ell_{R}$ and $\ell_{G}$ colours.

The separation of the four data-sets on the colour-diagram makes it possible to apply false colour enhancements (FCE) of the all-sky images. The procedure is introduced in our accompanied paper ([30]). FCE defines a colour shift to enhance the differences from the mean natural sky, linearly extending the colour range around a mean natural sky colour. There is no unique recipe for this method, and we selected a central point in CIE xy coordinates by trial and error to get a visually optimal colour difference. The colour space around this point is extended by a factor of two. The right panel of Figure 6 shows the all sky images after FCE processing. It clearly demonstrates that the procedure correctly enhances the colours to show the deviation from the normal sky conditions.

The only drawback of our colour diagrams is that the color shift caused by sodium enhancement is practically parallel with the shift resulted from ALAN dominated by sodium lamps. Therefore, it is not possible to distinguish sodium events from light pollution from a single measurement. However, continuous measurement at a given locations eliminates this problem. In addition, the structure of the sodium event on an all-sky image is very different from the structure defined by the light-domes of light pollution. Thus, data from even a single night can result in a clear cut between ALAN and sodium airglow, as it was the case at the Cosmic Campground Dark Sky Sanctuary.

To provide a practical demonstration of the defined colour metrics, we analysed the data obtained in our national park survey in Hungary. The work is still in progress. Therefore, the results provided here are only preliminary. Up to now, we performed measurements in six national parks and six additional protected lands, including the three Hungarian dark-sky parks. In some of the places, measurements were done at different dates. We selected a representative sample of 25 high-resolution all-sky measurements. We checked the statistics of the individual images first. Due to the light-domes of the cities, there is a gradient of radiance from zenith to horizon. In each image, we selected 1140 individual regions uniformly distributed on the sky. First, the extreme data points (e.g., affected by trees or other shading objects) were filtered out. Then, we binned the data in the G band band-averaged radiance. We used 0.4 dsu bands for the binning, to get enough data for all the samples. The mean and the standard deviation were calculated for all bins. The standard deviation of the $\ell_{R}$ and $\ell_{G}$ values are typically in the range of 0.002–0.01 and 0.0005–0.002, respectively. Those values represent well the range of colours for the given all-sky measurements. As expected, the radiance ($L_{G}$) correlates with the red colour $\ell_{R}$, but the colour change is relatively small. Typically, $\Delta L_{G}\approx \left(100-200\right)\Delta \ell_{R}$. The green colour ($\ell_{G}$) does not have any significant variation with the radiance. Therefore, for individual measurements, the $\ell_{R}-L_{G}$ plot provides important information.

**Figure 7:**
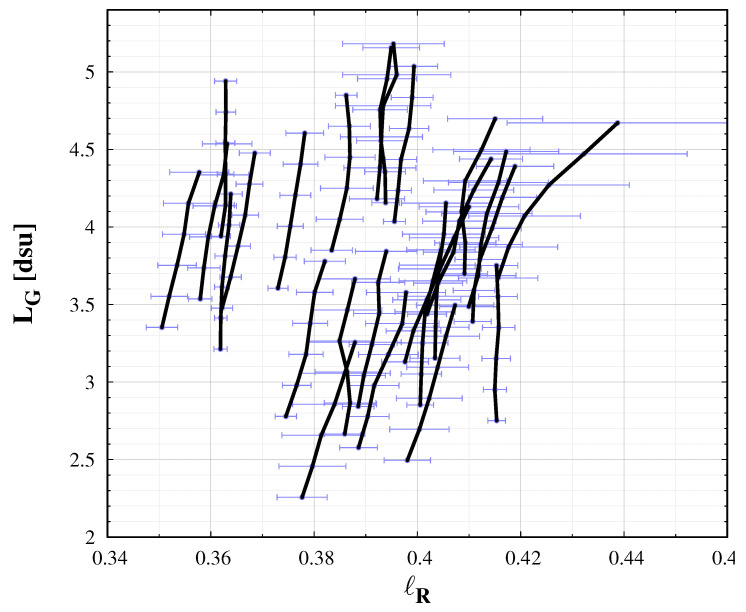
Colour–radiance diagram obtained from different locations in Hungary.

Figure 7 displays the $\ell_{R}-L_{G}$ distribution for all the measurements together. While the spread of $\ell_{R}$ in the individual measurements is typically ±0.005, the colour in all the measurements varies in the $0.35<\ell_{R}<0.42$ range. In our whole sample, we could not find any clear correlation between the colour and the radiance. This indicates that the colour of the illuminating sources is different at the different locations. We can rule out that different airglow levels play a major rule in colour variation, since the green colour is in a normal range ($\ell_{G}<0.35$). In addition, some of the measurements around the zenith shifted to the blue are correlated with extremely low atmospheric aerosol optical depths. It indicates that the colours at a given location can be a proxy for the aerosol optical depths. However, more data have to be collected in order to produce more definitive results on the effect of atmospheric conditions on colours.

The observations are represented by the triplet of the G radiance and two colours: ($\ell_{R}$, $\ell_{G}$, $L_{G}$). To check the independence of these data, we performed a principal component analysis (PCA) of our data sample. Here, we used the mean of the darkest regions only for each locations’ epoch. The normalized principal components are: 45%, 35% and 20%. It demonstrates that we need all three of these quantities to describe the quality of the night sky. Note that the PCA of the model (test) data presented in [30] gives very similar results, which indicates that the statistics of the model data represent the observations well. It is possible to introduce new variables based on the PCA by constructing uncorrelated data vectors. However, such new variables do not help in the interpretation of the data, thus we prefer to use the original $\ell_{R}$, $\ell_{G}$, $L_{G}$ triplets.

Adding special filters to the cameras system, for example, for the hydrogen and sodium lines independently, may improve the measurements at hardware level to reduce the effect of the short-term variation of natural sky radiance. However, such instruments with special filters would increase the cost of the measurements significantly. As such, this may not be practical for amateurs or citizen scientists, but may be an important asset for long-term night sky monitoring.

## 6. Materials and Methods

All the all-sky measurements presented in this paper were obtained by Sony ILCE 7SII cameras (Sony Digital Imaging, Chonburi, Thailand), equipped with Samyang 24 mm T1.5 VDSLR ED AS IF UMC II lens (Samyang Optics, Changwon, South Korea). The camera was attached to a robotic panorama head (GigaPan EPIC Pro, manufactured by GigaPan Systems, Portland, OR, USA). At a given location, we took 28 or 35 images with the motorized head to cover the whole sky with high resolution. The standard exposure setup for the measurements: ISO: 6400, exposure time: 6 s F/1.4 (T1.5) aperture. In the fixed all-sky camera monitoring stations we use the same camera body with Samyang 8 mm T3.8 VDSLR lens with ISO 6400, exposure time 30 s and F/3.5. The individual images are processed to calibrated radiance distributions of the sky in dsu units. Then, the images were stitched to each other to generate hemispheric images in spherical projection. All image processing besides the stitching of the images was performed by our DiCaLum library written in GNU Octave.

## 7. Conclusions

Commercial digital cameras provide an affordable solution to measure the radiance of the night sky and to determine sky quality. However, the natural changes in sky quality (e.g., because of the natural variation of airglow) jeopardize the interpretation of imaging radiometry. In this paper, we recommend a camera based colour-space which is suitable for separating the images with different radiation components. The normalized band-averaged radiances ($\ell_{R,G}$) provide an easy-to-use camera based colour system. Here, we summarize the major results:

- The correlated colour temperature (CCT) alone cannot distinguish all possible night sky colours.
- In the $\ell_{R}-\ell_{G}$ plot, the different sky colours are well separated.
- Small differences in colours can be enhanced by the false colour enhancement (FCE) method.

At the time of the submission of the manuscript, we have a suitable amount of data to define and test the methods. However, we are continuing our survey in Hungarian national parks and also some other locations. We expect to build a large database during the next few years, and we plan to refine the methods defined in this paper.

## Abbreviations

The following abbreviations are used in this manuscript:

dsu	Dark sky unit
FCE	False Colour Enhancement
